# New interactive machine learning tool for marine image analysis

**DOI:** 10.1098/rsos.231678

**Published:** 2024-05-22

**Authors:** H. Poppy Clark, Abraham George Smith, Daniel McKay Fletcher, Ann I. Larsson, Marcel Jaspars, Laurence H. De Clippele

**Affiliations:** ^1^ Marine Biodiscovery Centre, Department of Chemistry, University of Aberdeen, Aberdeen AB24 3UE, UK; ^2^ Department of Computer Science, University of Copenhagen, Copenhagen 2100, Denmark; ^3^ Rural Economy, Environment and Society, Scotland’s Rural College, Edinburgh EH9 3JG, UK; ^4^ Tjärnö Marine Laboratory, Department of Marine Sciences, University of Gothenburg, Sweden; ^5^ School of Biodiversity, One Health & Veterinary Medicine, University of Glasgow, Glasgow G61 1QH, UK

**Keywords:** marine image analysis, interactive machine learning, automated area measurement, RootPainter, benthic ecology, computer vision

## Abstract

Advancing imaging technologies are drastically increasing the rate of marine video and image data collection. Often these datasets are not analysed to their full potential as extracting information for multiple species is incredibly time-consuming. This study demonstrates the capability of the open-source interactive machine learning tool, RootPainter, to analyse large marine image datasets quickly and accurately. The ability of RootPainter to extract the presence and surface area of the cold-water coral reef associate sponge species, *Mycale lingua*, was tested in two datasets: 18 346 time-lapse images and 1420 remotely operated vehicle video frames. New corrective annotation metrics integrated with RootPainter allow objective assessment of when to stop model training and reduce the need for manual model validation. Three highly accurate *M. lingua* models were created using RootPainter, with an average dice score of 0.94 ± 0.06. Transfer learning aided the production of two of the models, increasing analysis efficiency from 6 to 16 times faster than manual annotation for time-lapse images. Surface area measurements were extracted from both datasets allowing future investigation of sponge behaviours and distributions. Moving forward, interactive machine learning tools and model sharing could dramatically increase image analysis speeds, collaborative research and our understanding of spatiotemporal patterns in biodiversity.

## 1. Introduction

Large image datasets enable detailed and long-term studies of underwater species, providing a vital tool for the ecological investigation of deep-water species [1–3]. However, extracting information of interest from image datasets, such as species presence or size, can be prohibitively time-consuming [4–6]. This problem is exacerbated for more complex images, such as those captured by mobile cameras on remotely operated vehicles (ROVs), or automated underwater vehicles (AUVs), where lighting and focus may vary compared to stationary underwater cameras or fixed observatories [7,8]. There has, therefore, been a trend to develop bespoke machine learning algorithms to extract information from a given image dataset, often as a result of collaboration between marine and computer scientists [4,9,10]. As machine learning algorithms are non-trivial to construct and apply, their accessibility to individuals without experience in scientific programming languages is limited. This creates a barrier to model sharing that is making some image analysis work redundant. Pre-developed, user-friendly and widely applicable machine learning tools may present a solution to some of these issues. They allow individuals with no machine learning or coding skills to develop models through training a pre-existing and adaptable ‘base’ neural network. The process to train models can vary depending on the tool employed but their complexity is often masked behind user interfaces.

RootPainter is one such user-friendly and open-source software tool, with a graphical user interface that enables the rapid training of convolutional neural networks via corrective annotation [11]. It is an interactive machine learning tool as the user is involved in the training process via a feedback loop; the algorithm presents successively improved predictions based on the users’ corrections in real-time. Users are not required to possess a graphics processing unit (GPU) with high computing power, or any coding competencies and models can be transferred between projects and users. RootPainter was initially developed to investigate root length and the presence of soil voids (bipores) in soil images with the production of a successful model being achievable within one working day. Internally, the tool uses a variant of the general-purpose U-Net convolutional neural network [12], chosen for its known competency with roots in soil [13]. U-Net has also demonstrated capabilities with marine objects, including fishes [14], coral reefs [15], demosponges [9] and sharks [16]. As U-Net introduces no requirements on the type of object that a model can be trained to detect, the application of RootPainter is not limited to soil images. That said, the innate complexity associated with marine images (due to suspended matter affecting image clarity and the uneven illumination of scenes with artificial lighting in the depths) may increase the time required to develop models of acceptable performance with RootPainter compared to image datasets from controlled (laboratory) conditions. Many automated marine image analysis workflows rely heavily on image pre-processing to diminish the complexity of their image datasets and improve model performance [9,17]. This can involve denoising or brightness/contrast/colour normalization and is one of the most time-consuming stages of the analysis pipeline [10,18], with the potential to limit model performance if the wrong augmentation is applied [19]. Reliance on non-trivial image pre-processing reduces both the accessibility of machine learning algorithms and the transferability of models, limiting processing pipelines to applications on specific datasets. It is, therefore, important that user-friendly machine learning tools, such as RootPainter, do not depend on user-controlled image pre-processing to produce successful models [20–23].

Increased accessibility and functionalities of machine learning tools will increase the rate and range of measurements that can be extracted from marine image data. RootPainter can simultaneously extract estimates of the perimeter, area, *x*,*y* coordinates, eccentricity and count of a given subject of interest within an image. These measurements have historically been manually acquired and used to draw ecological conclusions. For example, manual measurement of the length, perimeter or area of species from images has been used to estimate their size and growth rates [24–28], and extraction of count data has provided estimates of species abundance [29] and biodiversity [30]. Combined analyses have increased the value of information obtained further, allowing estimation of biomass through the extraction of both species’ abundance and individual areas [28,31,32], and investigation of sessile species behaviour through their size variation alongside local biotic or abiotic factors [33,34]. These measurements have, therefore, been key targets of marine machine learning studies. Multiple bespoke algorithms capable of automated marine species detection have been developed [4,35–37] but a few have published biodiversity estimates from these algorithms [38]. This may be the result of inherent difficulties associated with automated species detection, such as the need for each species to be annotated enough times in the training data to be detected when the model is subsequently applied [6]. The development of machine learning algorithms capable of predicting the area of sessile organisms from marine images has led to successful investigation of behaviour such as cold-water coral feeding [17,39] and sponge contractions [9,40,41]. Additionally, previously unknown species behaviour traits have been revealed by tracking individuals through extraction of their *x*,*y* coordinates along the sea-floor [2], and extraction of ‘global shape measures’ such as the eccentricity, or curvature, of individuals has allowed investigation of morphological diversity within or across species [42].

The ability to develop machine learning models capable of extracting measurements for multiple species at a time has the potential to further increase the efficiency of marine image analysis. However, this incurs inherent difficulties depending on varied species presence and visual complexity within datasets [43]. While the latter does not appear to have hindered benthic object detection models [4,35,37,44], there has been limited success with models capable of simultaneously, and differentially, extracting the area of multiple species [10,45]. When extracting deep-sea coral and sponge areas, Purser *et al*. found that the large variation in texture and colour of sponges at the site limited model performance; this may have been improved through increased exposure of the algorithm to sponges within the training dataset [10]. Recently, models performing simultaneous multi-species area estimations have been more successful [45], but their development is still dependent on non-trivial training and application of neural network architectures. Given these challenges, developing single species models, but with machine learning tools capable of extracting multiple measurements from image data, may provide an alternative accessible solution to increase the speed and complexity of ecological conclusions possible in benthic studies.

This study, therefore, investigates the suitability of RootPainter for marine image analysis. The potential of RootPainter to combat key challenges in the field was explored by testing its ability to identify and predict the surface area of a known difficult target for machine learning algorithms [10], the deep-sea sponge *Mycale lingua*. Additionally, RootPainter’s capabilities with images of varying complexity were assessed through a comparison of model performance for static time-lapse images from an underwater observatory, and frames extracted from ROV videos.

## 2. Material and methods

### 2.1. Study sites, underwater imagery and data availability

**Figure 1.**
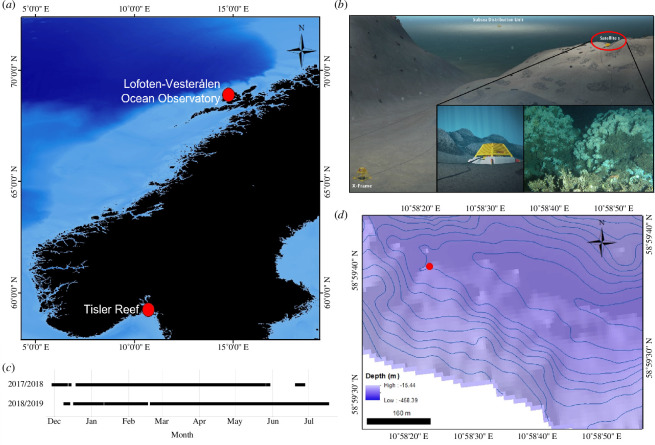
(***a***) Map of Norway highlighting the locations of the LoVe Ocean Observatory and Tisler reef. (***b***) Sub-sea layout of the LoVe Ocean Observatory including the satellite 1 structure responsible for collecting data used in this study and an example of the raw 5202 × 3464 pixel image output from satellite 1 during 2017−2019 (adapted from [46]). (***c***) Image data availability from the LoVe Observatory between 2017 and 2019. (***d***) Bathymetry map of the Tisler reef, with the 2021 ROV survey area pin-pointed with a red dot.

Data was used from two separate locations in Norway (figure 1*a*). Time-lapse imagery was captured at the cabled Lofoten Vesterålen (LoVe) Ocean Observatory at 240 m depth [46], and ROV videos were recorded at the Tisler reef, between 70 and 160 m depth [47].

The LoVe Observatory (68°54.474′ N, 15°23.14 E) is in the Hola trough, a continental slope 20 km from the Lofoten Islands [46]. Sub-station satellite 1 (figure 1*b*) was installed in 2017, supporting a Canon EOS 550 camera with E-TTL flash mode that captured 9173 hourly images throughout 2017, 2018 and 2019. Data was transferred through a total of 450 m of subsea cable, from the satellite to the central X-frame unit, where data from all sensors is collected, and finally to the observatory main cable at the subsea distribution unit (figure 1*b*; [46]). Observatory structure maintenance resulted in data gaps during this time (figure 1*c*).

The Tisler reef is found north of Tisler Island in a 48 km long ocean channel in the Hvaler area [7]. The research vessel Nereus, stationed at the Tjärnö Marine Laboratory was used to deploy the Ocean Modules ROV (V8 Sii, P/N: 02/00100-01, S/N: 011) to record videos on the eastern section of the Tisler reef in 2021 (figure 1*d*). A full-colour high definition Hama lens camera with two Bowtech LED-K-2400 lights (2400 lumens each) was used to collect the video footage. Video signals were transmitted over an optical fibre as the ROV moved. Two laser beams, separated by 5 cm, were used as a reference to scale video frames. An Applied Acoustics Nexus Lite USBL system, running the Applied Acoustics 1329A Micro beacon provided ROV navigation data. Every 130th frame was extracted from a total of 1 h and 55 min of video; this minimized content overlap between frames but maximized reef coverage (ROV speed varied during the survey). A total of 1420 images of 1920 × 1088 pixels were extracted as a result.

### 2.2. Target species

The Lofoten Vesterålen region and Tisler reef both host abundant *Desmophyllum pertusum* colonies (alternately known as *Lophelia pertusa*, Linnaeus 1758 [48]) and sponges, including *M. lingua* (Bowerbank, 1866). *Mycale lingua* was chosen as the target species to explore the capabilities of RootPainter as the complex range of colours, textures and morphologies that sponges display within a given species makes them difficult subjects for machine learning algorithms [10].

*Mycale lingua* (Bowerbank, 1866) is a Demospongiae found widely distributed across the northern hemisphere at depths of 30–2500 m, with particularly high concentrations in the North Atlantic Ocean [49]. *Mycale lingua* non-selectively consumes small (<10 µm) plankton [50] and is one of the only sponge species known to successfully colonize reef areas that have high *L. pertusa* densities [10,51,52]. Other than its association with cold-water coral reefs, little is known about the behaviour of *M. lingua*; thus far, there has been limited success in maintaining the sponge in aquaria for extended periods of time [53]. As sponges are important components of benthic ecosystems, both in the presence and absence of *L. pertusa* reefs [51,54], understanding their distribution, biomass and behaviour could allow evaluation of factors such as their contribution to carbon-cycling in benthic environments [31].

Using both the LoVe Ocean Observatory and Tisler reef datasets allows exploration of the ability of RootPainter to identify *M. lingua* from a more uniform dataset (i.e. one sponge in one location) and a more diverse dataset (i.e. different *M. lingua* individuals in different locations). In this study, adjoining sponge lobes were treated as one individual. *Mycale lingua* are known to exhibit lobed body structures [49] and without sampling it was not possible to confirm whether lobes were genetically distinct.

### 2.3. RootPainter

The software program RootPainter works through three stages:

- **stage 1:** users annotate images with clear examples until non-random model predictions are seen (requires 6–10 images);
- **stage 2:** users switch to corrective annotation, continuing to work through the training images which have been pre-segmented (images displaying predictions) by the current model. These corrections are included in the training data, continuously improving the model until users are satisfied with its performance, which is also indicated by multiple corrective annotation metrics; and
- **stage 3:** the trained model is then used to automatically process (segment) the full dataset.

The continuous feedback loop in stage 2 allows issues and anomalies to be addressed by the user that may have not been encountered in stage 1. This corrective annotation continually supplies measures of true and false, positives and negatives to the algorithm for each image. Multiple corrective annotation metrics (i.e. precision, recall, dice score and accuracy) can, therefore, be calculated during training without the need for separate manual annotations to validate the performance of the model. RootPainter (version 0.2.23 onward) can also estimate the error in the predicted surface area of the subject of interest by its models during training, allowing assessment of model success.

Once trained, models classify the pixels of each image into foreground and background, where the foreground represents the object of interest. These predictions are called segmentations; a visual output is provided for each image where segmentations are shown as blue highlighted regions. From these segmentations, six measurements can be simultaneously extracted by RootPainter, these include count and area of regions of interest, as well as the diameter, perimeter, the eccentricity of each discrete area and the *x*,*y* coordinates of the centroid of each discrete area. Eccentricity is computed based on an ellipse that has the second moments of the discrete region, it ranges from 0 to 1 and is the difference the approximated ellipse has from a perfect circle, with 0 meaning the ellipse is a circle. All measurements are computed using the scikit-image library [55]; when several subjects are present within one image, separate values for their finite areas, as predicted by RootPainter, are reported.

#### 2.3.1. RootPainter model training

RootPainter installation and model development were completed as per the GoogleColab notebook instructions [56]. A detailed manual describing model training, specifically for marine images, is also available [57].

**Figure 2.**
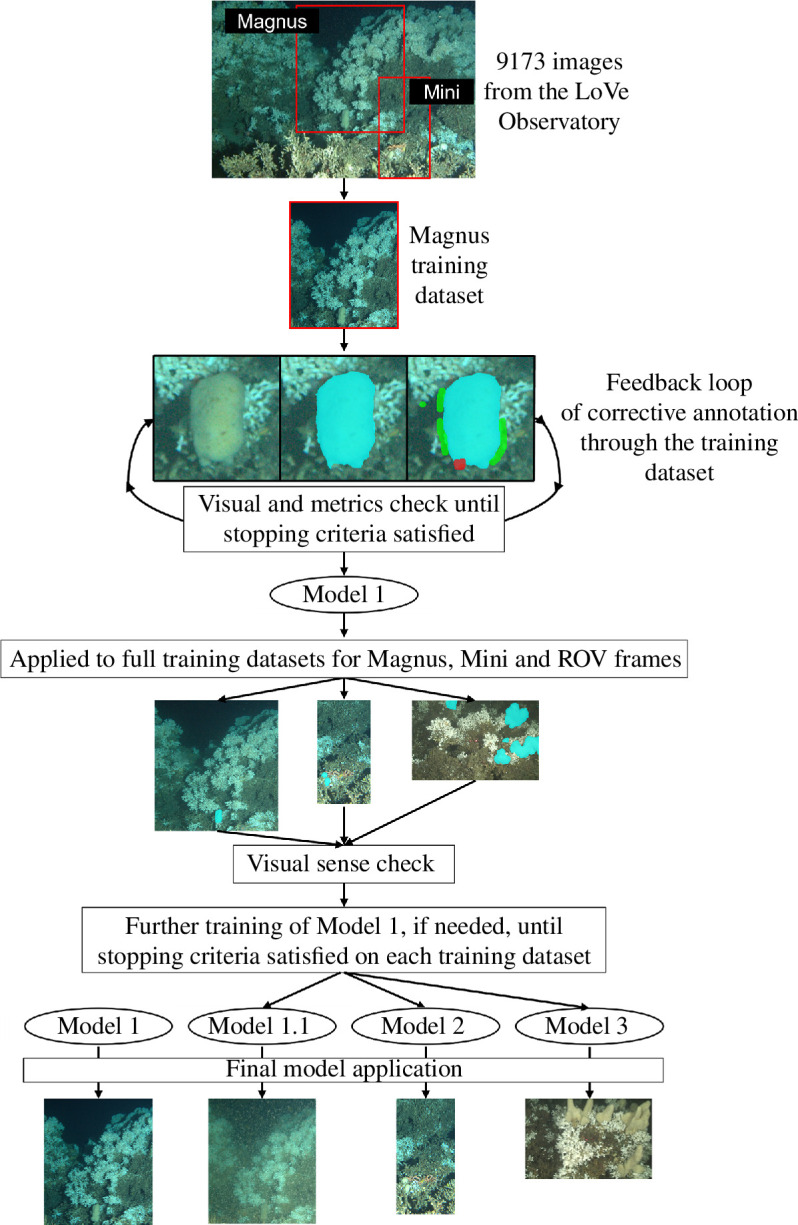
Model development workflow. Magnus and Mini from the LoVe Observatory were cropped into separate images, forming datasets of 9173 images each. The images of Magnus from 2019 were uploaded to Google Drive forming a training dataset. During RootPainter model training, the algorithm presented successive random images from the training dataset, along with its prediction for that image. The user then corrected this prediction, highlighting in green pixels that should be included background and in red pixels that should be included in the foreground. The continuous visual feedback loop and accompanying metrics allowed the determination of the endpoint of training, producing Model 1. This model was then applied to the Magnus, Mini and ROV frame datasets (the ROV images are shown at 1.5 times their true size, relative to the LoVe Observatory, for improved visualization). After checking the segmentation outputs, further model training was clearly required on April/May 2018/2019 for Magnus, and on images from 2018 for Mini; significant further training of Model 1 was required on the ROV frames. This additional training produced Models 1.1, 2 and 3. All four models were then applied to their total respective datasets.

The LoVe Observatory images were cropped using the ‘magick’ package [58], in R [59], to form two datasets containing 2200 × 2550 and 1000 × 1964 pixel images, each containing one *M. lingua* individual hereafter referred to as Magnus and Mini, respectively (figure 2). This allowed the evaluation of the efficiency of RootPainter on images of different sizes, as well as the model transfer function of RootPainter within a dataset. The ROV frames were cropped to 1400 × 888 pixels using the ‘magick’ package [58], in R [59], such that the lasers were centralized, the ROV display text was removed, and the far background of each was image limited (figure 2).

RootPainter was run through the free version of GoogleColab, with Google Drive used to sync image directories. To comply with free storage limits, 1 year of images from the LoVe Observatory were uploaded to Google Drive as the training datasets for Magnus and Mini; images of Magnus from 2019 and Mini from 2018 were used, as a shift in coral rubble obscures one of the lobes of Mini in 2019. The total ROV dataset of 1420 images was uploaded to Google Drive for use in training.

Once running, RootPainter presents random successive images to the user from the selected training dataset. Eight of these images were annotated with examples of foreground (species/substrate of interest, here *M. lingua*) and background (everything else in the image) before models provided non-random predictions. Subsequent segmentations were then corrected, highlighting false positives (overpredictions that should be background) in green and false negatives (underpredictions by the current model) in red (figure 2). These corrections were incorporated into successive new and improved models. All corrective annotations are automatically saved within the user’s Google Drive project folder. In order to comply with the free GoogleColab GPU usage limits, annotations in this study were completed in 3–5 h sessions.

In total, five RootPainter models were produced [60]. Model 1 was developed on images of Magnus. Additional fine-tuning of Model 1 was required on images of Magnus from April/May of 2018/2019 due to a change in sponge colour/texture and turbid conditions; Model 1.1 was then applied on images from this time and Model 1 to the remaining images of Magnus. Model 1 was also transferred and served as a training starting point on images of Mini and *M. lingua* within ROV video frames, producing Models 2 and 3, respectively (figure 2). Model 2 was then applied to a Google Drive folder that contained all 9173 images of Mini, and Model 3 to a folder containing all 1420 ROV video frames. Model 4 was trained to identify the lasers in the same 1420 ROV frames from the Tisler reef, cropped to 1400 × 888 pixels, independently of all other models. Inter-observer variation was avoided as the same individual completed the training of all models.

#### 2.3.2. Stopping criteria

Two distinct approaches were used to determine the endpoint of model training. Training cessation was guided by qualitative criteria for Models 1–3 but quantitative criteria only for Model 4. The success of all models was also quantitatively assessed in real-time using the corrective annotation metrics of precision, recall, dice score, accuracy and estimated area error. This permitted exploration of the suitability of corrective annotation metrics to use as stopping criteria in future studies, without compromising the integrity of model results. Training was deemed complete for Models 1 and 2 (LoVe Observatory) when predictions for at least two images from each month of the training dataset had required no corrective annotation. For Model 3 (ROV Tisler), segmentations that did not require any corrective annotation had to be seen for three frames from each video section; this more stringent criterion reflects the higher variability of image content and quality in the Tisler dataset. For Model 4 (ROV Tisler), the simplicity of the subject of interest and its stark contrast to any background objects permitted the decision to stop training to be solely determined through RootPainter’s metrics calculations; specifically, when the rolling average (*n* = 10) for the dice score reached 0.95.

#### 2.3.3. Post-processing

Application of trained models to their respective datasets, via the ‘segment folder’ function in RootPainter, produced foreground predictions for every image. The discrete area values of each foreground prediction were extracted using the RootPainter ‘extract region properties’ function and exported as one .csv file.

The results of Models 1 and 2 (LoVe Observatory) and Model 4 (ROV Tisler) were visually checked for anomalies. This involved scanning through the segmentation output file thumbnails for obvious errors, such as camera malfunctions, missing sponge/laser areas or obstructions by fishes. This facilitated faster and more comprehensive data point exclusion than attempting to identify anomalies through pre-processing; post-processing required approximately 30 min of active work per 744 images analysed. Significant variation in sponge area and distribution in Tisler reef video frames prevented identification of Model 3 errors through segmentation observation alone. Given that additional approaches, such as direct visual comparison between RootPainter segmentations and their respective input images, would suffer diminishing returns for an increase in result accuracy with user time, no post-processing was conducted for results from Model 3.

### 2.4. Image scaling

#### 2.4.1. Images from the Lofoten Vesterålen Observatory

In the absence of laser scales, the average width of an *L. pertusa* branch from the Bømla reef in Hardangerfjord (0.43 cm ± 0.09 [15,61]) and branches adjacent to Magnus and Mini were used to scale the pixel dimensions of images in ImageJ [62]. This allowed the conversion of the foreground areas predicted by RootPainter from pixels to cm^2^. Relative sponge areas were calculated through the division of each surface area value by the maximum sponge area value for that dataset.

#### 2.4.2. Remotely operated vehicle frames from the Tisler reef

The *x*,*y* coordinates of areas segmented by Model 4 allowed calculation of the distance between laser points in each ROV video frame in pixels (equation (2.1)). Images were then independently scaled based on the true distance between the lasers, which is 5 cm. The area errors for each image, as calculated by RootPainter during training, were scaled in the same manner.


(2.1) $$distance\;between\;lasers\;(pixels)=\sqrt{{\left((x_{1}-x_{2}\right)}^{2}+(y_{1}-y_{2}{)}^{2}}$$

Equation 2.1: formula used to calculate the Euclidean distance between laser points given their *x*,*y* coordinates, where (*x*_1_,*y*_1_) and (*x*_2_,*y*_2_) correspond to each laser point respectively.

### 2.5. Model validation and statistical analysis

Model 1 was validated by comparing surface area measurements made manually in Photoshop [63] and predicted by RootPainter for 452 images (5% of the total dataset), randomly selected using an R script [59]; 28 of which were seen during training. Sponge areas were extracted from the Photoshop annotations using open-source R scripts [63] and scaled using ImageJ [62], as previously described. The precision, recall, dice score and accuracy of Model 1 were then calculated in Python [60,64], by assuming the manually annotated images were accurate.

Precision is quantified as the ratio of true positives to all positive instances (the sum of true and false positives) and describes the probability that a pixel is truly foreground, given that the RootPainter model predicts it as foreground. Recall is calculated as the ratio of true positives to all true positive instances (the sum of true positives and false negatives) giving a measure of the proportion of foreground pixels the RootPainter model is expected to identify [65,66]. Dice score is calculated using precision and recall, giving an overall indication of model performance. Accuracy evaluates how close the true result is to the model’s predictions based on the degree of overlap between predicted segmentations and the true regions [66]. In previous studies, models have been defined as successful with a precision ≥0.71, recall ≥0.75, dice score ≥0.74 and accuracy ≥0.76 [4,9,17,35].

RootPainter also continually calculates these metrics during training but through the assumption that corrected segmentations are accurate. Comparison of the corrective annotation metrics for Model 1 to the externally calculated validation metrics allowed evaluation of the necessity of separate manual validation for future RootPainter studies. Additionally, RootPainter provides estimates of area error during training allowing assessment of model success. Error is calculated through subtraction of the ‘corrected area’ from the ‘predicted area’ for each training image, where the corrected area is the post-annotation result, and is taken to be the true area. For Model 3 the agreement between the corrected area and RootPainter’s training prediction was also investigated through the calculation of a Pearson correlation coefficient and linear regression, to ensure a lack of bias across multiple sponge individuals of varying sizes.

## 3. Results

### 3.1. RootPainter model development

In total, three models were produced and used to evaluate the surface area of *M. lingua*; a fourth model was produced to identify red lasers in ROV video frames. Table 1 displays the number of images and time used in both training and application of the models.

**Table 1.** Training and application data for RootPainter Models 1, 2, 3 and 4. (Additional learning time refers to time connected to GPU where no annotations were performed but training was left running to enable the model to better fit the existing annotations. The total images for Magnus include 120 additional images used to optimize Model 1 to turbid images during a colour/texture change in April/May (figure 2; electronic supplementary material, table S1).)

RootPainter model	1	2	3	4
subject of interest	Magnus	Mini	*Mycale lingua*	lasers
image source	LoVe Observatory	LoVe Observatory	Tisler reef ROV	Tisler reef ROV
training				
dataset	2019 and April/May 2018	2018	2021 Tisler East	2021 Tisler East
total images correctively annotated	640	142	556	100
corrective annotation time (hours)	17	3.5	10.5	0.75
additional learning time (hours)	8	3	0	0
application				
dataset	2017/2018/2019	2017/2018/2019	2021 Tisler East	2021 Tisler East
total images segmented	9173	9173	1420	1420
segmentation time per image (seconds)	10.7	3.0	1.1	1.1

### 3.1.1 Model 1

**Figure 3.**
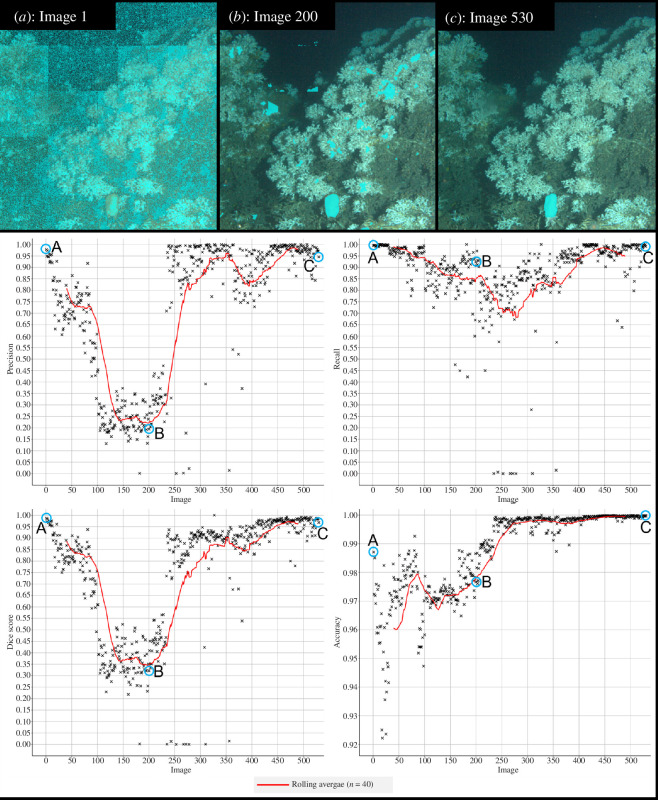
RootPainter predictions for images that appeared 1st, 200th and 530th during training of Model 1, where segmentation by the model is shown in light blue overlaying the input image. Accompanying corrective annotation metrics graphs display changes in precision, recall, dice score and accuracy of Model 1, as calculated by RootPainter during training, with the values for the 1st, 200th and 530th images labelled as A, B and C respectively. Values are displayed until image 530; the additional 120 images used in training RootPainter to recognize Magnus (table 1), developed Model 1.1 (electronic supplementary material, figures S1 and S2).

Model 1 was trained on 640 images of Magnus from the LoVe Observatory, requiring 17 h. The training times for Model 1.1 (electronic supplementary material, table S1), necessitated by the colour/texture change in Magnus during April and May of 2018/2019, are incorporated into Model 1 in table 1. The decision to stop training Model 1 was guided by qualitative criteria, but concurrent increases in the corrective annotation metrics of precision, recall, dice score and accuracy can be seen with improved segmentations in figure 3.

Model 1 was applied to 9173 images of Magnus. Post-processing to exclude anomalies was completed and highlighted that segmentations were impacted during March 2019, when sea-stars (suspected *Henricia* spp.) took prolonged residence on the base of Magnus. The area of sponge covered by the sea-stars varied, preventing reliable data point exclusion. Thus, segmentations from this period should be interpreted with caution. In total, 548 data points were excluded with 352 of these corresponding to corrupted images.

**Figure 4.**
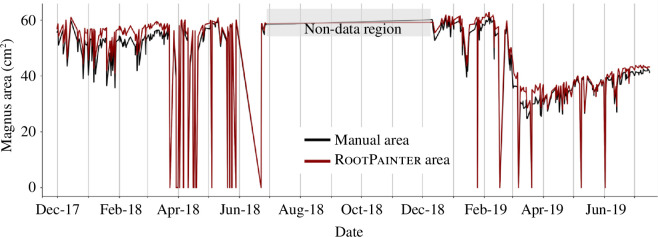
Comparison of Magnus’ area values as predicted by RootPainter and measured manually in Photoshop. Area highlighted in grey represents period during which no image data was available from the LoVe Observatory.

Figure 4 visualizes the agreement between the areas of Magnus extracted using Model 1 and those manually measured in Photoshop. The average difference in area values between the methods is 2.26 ± 1.69 cm^2^ or 5.3 ± 3.0% of Magnus.

### 3.1.2 Model 2

Fine-tuning of Model 1, through further training, was needed to produce Model 2 due to the differential lighting of Magnus and Mini at the LoVe Observatory. This required an additional 142 images and 3.5 h of corrective annotation on images of Mini, with the decision to stop training guided by qualitative criteria. The corrective annotation metrics from Model 2 training can be seen in the electronic supplementary material, figure S3. Model 2 was applied to 9173 images of Mini and post-processing was completed to identify anomalies. In total, 601 data points were excluded, with 352 of these corresponding to corrupted images.

### 3.1.3. Model 3

**Figure 5.**
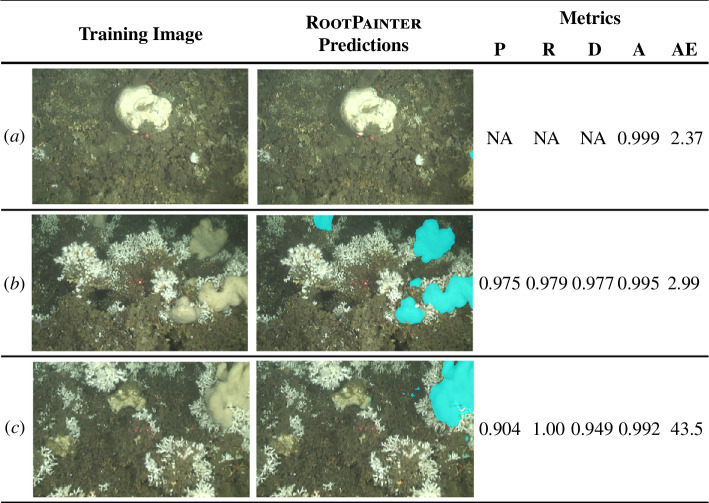
Examples of successful segmentations by RootPainter Model 3 and their accompanying metrics, where P, precision; R, recall; D, dice score; A, accuracy; and AE, area error (cm^2^). The images show; (*a*) *Geodia* spp. that is not misidentified as *M. lingua*, (*b*) *M. lingua* individuals accurately segmented within *L. pertusa,* and (*c*) *M. lingua* segmented accurately with nearby *Geodia* spp.

Fine-tuning of Model 1 to produce Model 3 was necessary due to the more complex and changing nature of ROV video frames compared with underwater observatory images. This required 10.5 h of corrective annotation on 556 video frames from the east of the Tisler reef, captured in 2021. The decision to stop training was guided by qualitative criteria, but the agreement between visual observations and the RootPainter corrective annotation metrics for Model 3 is demonstrated in figure 5. Model 3 can distinguish *M. lingua* from *L. pertusa* (figure 5*b,c*) and the sponge *Geodia* spp. (figure 5*a*,*c*).

Model 3 was applied to all 1420 ROV video frames from the east of the Tisler reef, captured in 2021. No post-processing was completed on the results from Model 3.

### 3.1.4. Model 4

Model 4 was developed to segment red ROV lasers. It was trained on 100 video frames from the east of the Tisler reef, captured in 2021, requiring 45 min. The termination of training was solely determined by RootPainter’s metric calculations (electronic supplementary material, figure S4). Model 4 was applied to all 1420 ROV video frames from the east of the Tisler reef, captured in 2021. Post-processing resulted in exclusion of 124 data points where only one laser was present.

### 3.2. RootPainter model performance

#### 3.2.1. Efficiency

RootPainter was 5–16 times more efficient compared to manual annotations (table 2). Using RootPainter to analyse an ROV dataset requiring multiple annotations per image was more efficient than manual annotation of an underwater observatory dataset containing one individual per image (Magnus).

**Table 2.** Analysis time in seconds per image for Manual and RootPainter methods. (Active user time for the manual method only includes the annotation times; for RootPainter, it is the corrective annotation times and post-processing times combined. Inactive time for the manual method only includes the image area extraction time in R; for RootPainter, this is the additional learning time and segmentation times (area extraction times were negligible for RootPainter). Magnus and Mini are both *M. lingua* individuals.)

subject of interest	annotation method	image source	active user time (s per image analysed)	inactive user time (s per image analysed)	total time required (s per image analysed)
Magnus	manual	underwater observatory	105	26.0	131
Magnus	RootPainter	underwater observatory	9.09	13.8	22.9
Mini	RootPainter	underwater observatory	3.80	4.18	7.98
*M. lingua*	RootPainter	ROV	26.7	1.10	27.7

#### 3.2.2. Accuracy

The precision, recall, dice score and accuracy for Model 1 are displayed in table 3; agreement between the metrics as calculated by external manual validation and internal training calculations in RootPainter can be seen. Average corrective annotation metrics from the endpoint of training of Models 2–4 can be seen in the electronic supplementary material, table S3.

**Table 3.** Performance metrics for Model 1. (Values from manual validation were calculated as a total result of overlaying all 452 manual annotations and their corresponding RootPainter predictions, meaning the calculation of a standard deviation is not possible.)

model	calculation source	precision	recall	dice score	accuracy	training images used to calculate average
1	manual validation	0.95	0.92	0.94	1.00	na
1	RootPainter corrective metrics	0.97 ± 0.04	0.96 ± 0.06	0.96 ± 0.03	1.00 ± 0.00	430–530

#### 3.2.3. Assessment of model success

**Figure 6.**
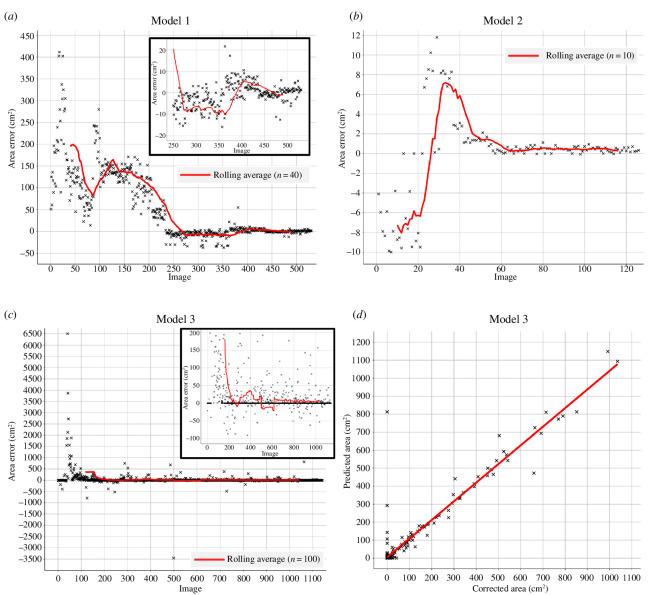
Graphs displaying changes in scaled area errors during training of: (***a***) Model 1, (***b***) Model 2, and (***c***) Model 3. (***d***) Graph demonstrating the correlation between *M. lingua* surface area as predicted by RootPainter and corrected during training for images 730–1130. The predicted area consists of all pixels RootPainter classified as *M. lingua* for each training image. The corrected area consists of all the pixels RootPainter classified as *M. lingua*, minus those the user highlights in green and plus additional pixels the user highlights in red.

Precision, recall, dice score and accuracy can reflect disproportionately harshly on model performance when foreground pixels are low (electronic supplementary material, figures S5 and S6). These corrective annotation metrics were, therefore, used in combination with the area errors as calculated by RootPainter to assess the success of Models 1–3 (figure 6). The agreement between the corrected/true area in each training image and RootPainter’s training prediction was also assessed for Model 3, as individuals of varying sizes are present in the ROV video frames (figure 6*d*). For the final 400 images used in training, the Pearson correlation coefficient between the corrected area and predicted RootPainter area is 0.95 (*p*‐value <2.2 × 10^-6^); Model 3 consistently over-predicts the area of *M. lingua* by 4.89 cm^2^ as calculated by linear regression, with an *R* ^2^ of 0.91.

The average area errors for each model, as calculated by RootPainter, towards the end of training can be seen in table 4. The value for Model 1 is in agreement with the average area error calculated from manual validation (2.26 ± 1.69 cm^2^, figure 4).

**Table 4.** Average area errors for Models 1, 2 and 3 as calculated by RootPainter during training. Average area error as a percentage was calculated using the average size of Magnus in 2019 for Model 1, and Mini in 2018 for Model 2 as this was the data used in training the models. The percentage area error cannot be accurately estimated for Model 3 due to the wide range of sponge sizes within the data.

model	average area error		training images used to calculate average
	cm^2^	%	
1	−0.06 ± 2.87	0.14 ± 6.67	430–530
2	0.45 ± 0.86	4.05 ± 7.75	100–150
3	7.09 ± 52.97	NA	730–1130

### 3.3. Model outputs and observations

In total, four measurements were simultaneously extracted by RootPainter from the output segmentations of Models 1–3, including the area of individuals, as well as the diameter, perimeter and *x*,*y* coordinates of each discrete area. For the purposes of this study, we focused on the surface area outputs only.

In the LoVe Observatory dataset, 100% of the images contained the target species, *M. lingua*. The average two-dimensional surface area for Magnus and Mini in the monitored months of 2018/2019 is displayed in table 5. In the Tisler reef ROV dataset, only 40% of the extracted video frames contained *M. lingua* individuals, with an average size of 19.4 ± 51.8 cm^2^.

**Table 5.** Average two-dimensional size of Magnus and Mini at the LoVe Observatory in the monitored months of 2018/2019.

year	average two-dimensional sponge surface area (cm^2^)	
	Magnus	Mini
2018	59.9 ± 3.8	11.1 ± 2.2
2019	43.0 ± 9.8	6.1 ± 0.8

**Figure 7.**
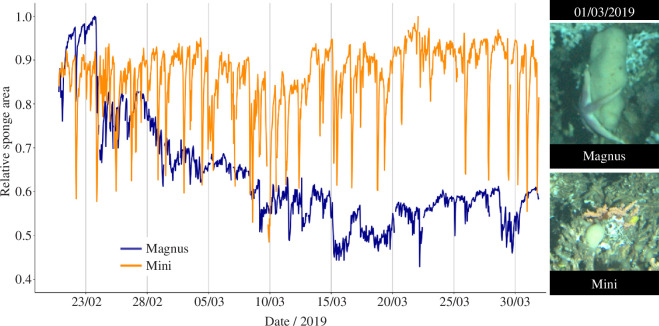
Relative areas of Magnus and Mini while sea-stars reside on the base of Magnus.

Magnus and Mini both exhibited frequent contractions in each month of recorded data, without any displayed seasonality in this behaviour. A clear decrease in sponge surface area (approx. 50%) in the results from Model 1 compared with Model 2 was seen during February–March of 2019. Returning to the raw data revealed that this resulted from prolonged sea-star (*Henricia* spp.) residence and presumed predation at the base of Magnus. Mini’s area was unaffected during this time (figure 7).

## 4. Discussion

This study showed the suitability of the user-friendly machine learning tool, RootPainter, to analyse large datasets of marine images. RootPainter was capable of accurately processing images of varying size, colour, and complexity, 5–16 times faster than manual annotation, without the need for image pre-processing. As the efficiency of RootPainter is dependent on dataset size, this may be even faster for larger datasets. Manual validation demonstrated the reliability of the qualitative stopping criteria used and RootPainter’s in-built metrics calculator as a means to assess model success. Therefore, external stopping criteria and model validation may not be required in future studies, allowing ecological conclusions to be drawn, with the appropriate caveats in place, with significantly improved efficiency.

### 4.1. Machine learning tools for marine image analysis

This work demonstrates that RootPainter is an accessible and affordable tool capable of processing large and complex datasets, with the potential to ease the analysis bottleneck created by the continually increasing volume of video/image data collected by marine researchers. The intuitive interface and instruction notebook accompanying the software allows marine experts to concentrate on dataset content, instead of the intricacies of running a machine learning algorithm. This includes removing the need for image ‘pre-processing’ steps, such as reduction of background complexity, as seen for other machine learning methods. The ability to run RootPainter through GoogleColab prevents users from needing to acquire an expensive GPU or to possess significant computing power. Consequently, powerful results were produced with no previous user experience in machine learning and at no additional cost.

This study focused on RootPainter’s application to *M. lingua* individuals only. However, the success demonstrated with this notoriously complex species of interest, paired with previous terrestrial examples of model success [11,67–69], gives confidence that RootPainter will be capable of segmenting other marine species. The applicability of RootPainter to species identification and biodiversity investigations may be increased by the introduction of multi-annotation capabilities. Within the current version of RootPainter, simultaneous investigation of multiple species requires the development of several separate models for each target species (i.e. class) and the extraction of results separately. Alternatively, a multi-staged approach can be used where the general foreground is segmented first and then this is used to remove all background from the data. The extracted foreground could then be further categorized into different classes. While this model-cascade approach may have training and efficiency benefits, akin to localization [70], its use decreases with increasing class number. That said, using a single species model approach may lead to more effective and accurate training by the user, through simplification of the training process and pattern recognition required of them. As segmentation and annotation files in RootPainter are ordinary PNG files, they can easily be copied out of the project and used with other software, for example, to train other neural networks. It is also possible to assign the user’s corrective annotations to segmentations created during training, and thus download the final corrected masks, allowing integration with other marine image analysis workflows at each stage of the model development process. This is of increasing importance as the marine image analysis community strives towards standardization of image annotations.

RootPainter is not the only tool that exists to automatically annotate marine images. The web-based annotation software BIIGLE is widely used by marine ecologists for manual annotations and is capable of automated novelty detection [71]. The user-friendly and open-source ‘machine learning-assisted image annotation’ (MAIA) function in BIIGLE has proved suited to biodiversity studies due to its multiclass annotation capabilities [37]. However, compared to RootPainter and at the time of writing (March 2024), extraction of information such as species area or perimeter cannot be automated in BIIGLE, no training metrics are provided to aid assessment of model success, and models developed through the MAIA function cannot currently be transferred between users through the existing interface.

The software ImageJ is also widely used by marine researchers for manual image analysis [62]. While it does not possess its own machine learning tool as such, the deepImageJ plugin enables users to apply pre-trained neural networks (models) in ImageJ, that are downloadable from an ‘online zoo’ [72]. The range of models that can be downloaded and information that can be extracted with them is extensive. Thus far, deepImageJ has been targeted at microscopy work and biomedical imaging, such as virtual tissue staining [73] and instance segmentation of neurons [74]. The accessibility of model sharing and application within deepImageJ has undoubtedly made significant progress in unifying the field of microscopy image analysis [72], with some models being downloaded 20 000 times [75]. In contrast to RootPainter, deepImageJ does not possess *de novo* model development or continued training capabilities. As generating models externally, or through additional plugins, requires machine learning expertise this creates a dependence of non-experienced users on others to develop models they require. When analysing marine images, the inability to optimize models without computational expertise would act as a significant barrier to the use of deepImageJ, as the quality and background of underwater images vary significantly.

There are user-friendly machine learning tools capable of extracting area measurements for subjects of interest other than RootPainter available. These include but are not limited to Tator [76], CVAT [77] and Biodoc [78], which all possess in-house annotation and automated segmentation capabilities without the need for image pre-processing. These tools can be run through cloud-based computing and allow users to export their annotations and output segmentation masks, facilitating their integration with other image analysis tools. Depending on the size and duration of a project Tator and CVAT may require users to upgrade their access through paywalls. Alternatively, the convolutional neural network termed ‘You Only Look Once’ (YOLO) [79] is also capable of automated segmentation and is becoming increasingly user-friendly through the production of thorough GoogleColab notebooks [80,81] and the development of accompanying graphical user interfaces [82,83]. Similarly to RootPainter, YOLO can automatically apply data augmentation to enhance model performance [44], so non-trivial user-controlled image pre-processing is not required [84]. Users are encouraged to begin training using one of YOLO’s pre-trained models, but *de novo* development is possible. As YOLO does not possess annotation capabilities, the images used in training must have been previously labelled using a separate software. The capability of YOLO to automatically detect objects from marine images has been shown; YOLO version 4 [85] was used to develop a model to identify the Xenophyophore, *Syringammina fragilissima* (Brady, 1883), within 58 000 AUV video frames, requiring less than 10 days for complete analysis, and achieving a final precision of 0.91 and recall of 0.84 [86]. Additionally, the recently released YOLO version 8 [87] has been used to develop a model to simultaneously quantify the coral *Dendrophyllia cornigera* [44] and sponge *Phakellia ventilabrum* [44] within 5201 transect images [44]. The fully trained YOLOv8 model required just over 2 h to process the data, with detection metrics depending on species and study sight, but all surpassing 0.85 [44]. As the multiclass annotation and transfer learning capabilities of YOLO (version 4) have also been demonstrated [88–90], model sharing and optimization with this algorithm may serve to ease the image analysis bottleneck for future studies of marine species’ distributions and biodiversity.

Compared to RootPainter, using any of the aforementioned tools requires images to be annotated before the initiation of model training. Supplied annotations are then used by the tools to complete training in the absence of user input. This passive training process may compensate for the additional time required by users to create a manually annotated training dataset but may also limit the extent of model optimization possible compared to a human-in-the-loop approach, such as with RootPainter [91]. As all software has advantages and disadvantages depending on user needs, a conscious decision regarding choice of machine learning tool for a desired investigation needs to be made. Machine learning tools that use graphical interfaces to increase their accessibility, like RootPainter, can demonstrate reduced flexibility as only pre-programmed instructions are executable [92]. Graphical user interfaces can also be slower and require higher storage space than interfaces based only on command lines, which may become limiting when moving to extremely large datasets. This has not hindered RootPainter studies so far, but further investigations with larger datasets (>20 000 images) are required. Users should also consider the dependence of cloud-based computing on a stable internet connection, and whether their machine learning tool of choice can also be run locally for applications in the field.

Overall, this work has demonstrated the suitability of RootPainter to marine image analysis studies. The tool has demonstrated a successful compromise between accessibility and algorithm flexibility through its graphical user interface and ability to be run locally or through cloud-based computing. The interactive training process and corrective annotation metrics facilitate fine-tuning of models and real-time assessment of model performance in a unique manner. These functionalities may also serve to aid studies based on models developed using other machine learning tools. In this way, and with improved integration between user-friendly machine learning tools, RootPainter may allow a model zoo to achieve similar success to that seen with deepImageJ, but for the marine field.

### 4.2. RootPainter model sharing

Sharing RootPainter models may allow researchers to dramatically increase their marine image analysis capacity and analyse datasets to their full potential. However, the time-saving capabilities of transferred models within RootPainter probably depend on the specific task and datasets used [93]. Starting training with a suitable pre-established model may reduce the time and number of images required to produce a satisfactory model for a given dataset; only 3.5 h and 142 images, and 10.5 h and 556 images were required to optimize Model 1 to produce Models 2 and 3, respectively (table 1). The initial increased accuracy of RootPainter predictions, as seen for the first 20 images of Model 2 compared to Model 1 (figure 3; electronic supplementary material, figure S4), significantly reduces the corrective annotation time required per image (table 2). Pre-developed models can also reduce the threshold number of ‘application images’ at which using RootPainter becomes more efficient than manual annotation. For *de novo* model development on static observatory images a minimum of 468 images are needed in the application dataset to ‘justify’ the use of RootPainter. Using a pre-developed model reduces this to 96 static observatory images or 289 ROV video frames (tables 1 and 2). However, greater image numbers may be required depending on the dataset (table 1).

The accuracy of transferred models will always be limited by variations between datasets. Researchers are, therefore, advised to use the adaptability of the RootPainter algorithm to fine-tune a pre-developed model to their dataset before its application [94]. This will also produce corrective annotation metrics, allowing users to assess the success of segmentations themselves. The ability to fine-tune models within the current version of RootPainter is limited to models trained using the base U-Net implementation. Users can edit the source code of RootPainter to allow fine-tuning of models trained using another neural network architecture, but a user-friendly way to achieve this does not yet exist within the RootPainter interface. Recently, the landscape of available pre-trained vision models has changed substantially, with large foundation models such as 'Segment Anything Model' now performing well on a variety of tasks out of the box [95], with marine image datasets, such as ‘TrashCan’ [96], already included in the training data. Obtaining suitable performance from these large pre-trained models on novel datasets can still require non-trivial fine-tuning [97]. Therefore, facilitating users’ ability to switch model architectures within the RootPainter interface may provide an accessible way to quickly adapt these large pre-trained models to a broader variety of marine datasets. Future studies will investigate the fine-tuning of large pre-trained models with RootPainter and further expansion of model sharing options for the marine image analysis community.

### 4.3. Analysing static versus mobile image datasets with RootPainter

#### 4.3.1. Dataset effect on speed of training

Image analysis with RootPainter is highly efficient for both static images and frames from moving videos (table 2). However, the number of images and training time required for model development on a given species does increase when moving from underwater observatory images (Models 1 and 2) to ROV video frames (Model 3). The more dynamic background, reduced image clarity and varied lighting within the ROV video frames, as well as the need to identify and distinguish many different *M. lingua* individuals from apparently similar *Geodia* spp., increased the extent of model optimization required to produce Model 3 compared to Model 2 (table 1).

Interestingly, the rate of corrective annotation in RootPainter did not decrease with increasing image complexity; optimization of Models 2 and 3 did not involve significant background annotations, with both requiring 1.2 min of annotation per image (table 1). Conversely, the rate of manual annotations does decrease with increasing image complexity (i.e. more individuals per image require more time to manually annotate). Therefore, a comparison of the development speed of Model 3 to underwater observatory manual annotations probably underestimates the efficiency of RootPainter for ROV video frame analysis.

It is important to note that the nature of the subject of interest also impacts RootPainter model training time. As the red lasers were uniform in each image and visually distinct from all other background objects development of Model 4 required the least time and number of images, despite being trained on ROV video frames.

#### 4.3.2. Dataset effect on accuracy of models

All RootPainter models in this study exhibited high levels of accuracy (table 3; electronic supplementary material, table S3). Manual validation confirmed that Model 1 consistently and accurately predicted the area of Magnus (table 3; figure 4). Poor agreement between the Photoshop and RootPainter results was often a result of external factors, such as sea-star presence or turbidity (electronic supplementary material, figure S7). The average area error of Model 1, as calculated by manual validation, is larger (5.3 ± 3.0%) than the estimate provided during training by RootPainter (0.14 ± 6.67%), but not significantly so. Therefore, we may accept the average area error estimates for Models 2 and 3 calculated by RootPainter during training (0.45 ± 0.86 cm^2^ and 7.09 ± 52.97 cm^2^, respectively), to be representative of the true accuracy of area predictions for these models (table 4).

The accuracy of Models 1–3 can be seen to decrease with increasing dataset complexity. Model 1 was trained on, and used to segment, images of the same individual. Conversely, each image segmented by Model 3 contained different sponge individuals, including many that the model was not exposed to during training. The effect of this is apparent in the larger average area error for Model 3 than for Model 1 (table 4). However, it should be considered that the accuracy of manual annotations may also decrease across these two datasets, and the overall area error for Model 3 is still acceptably small.

### 4.4. Increasing the efficiency of RootPainter

The efficiency of image analysis with RootPainter depends on the images and computing set-up used. Without non-trivial pre-processing to reduce image complexity, the main methods to increase analysis efficiency involve using smaller images, paying for upgraded GoogleColab access, or investing in a purpose-built deep-learning workstation. As the aim of this tool is to be accessible and cost-effective, the use of equipment designed for deep-learning will not be discussed further here.

Smaller images increase the efficiency of image analysis with RootPainter through increased training speeds and reduced application times [11]. When constrained to larger images, using the ‘create dataset’ function in RootPainter to randomly crop training images can produce a more efficient training dataset [11]. Smaller images require less time to segment during model application; Model 2 was applied to images 2.8 times smaller than Model 1 (figure 2), and they were segmented 3.6 times faster (table 1). The same application segmentation speeds seen for Models 3 and 4 (table 1), demonstrate that subject complexity does not affect RootPainter application time.

Upgrading GoogleColab can significantly reduce both the ‘active’ and ‘inactive’ user time required for RootPainter studies, through increased access to higher memory GPUs. Chance assignment to a higher memory GPU resulted in reduced segmentation times during the application of Models 3 and 4 compared with Model 2 (table 1), despite their application to images of similar sizes. The application efficiency of RootPainter may, therefore, be tripled if improved GPU assignments can be consistently secured through a paid upgrade in GoogleColab (approx. £10 a month in the year 2024). As access to the paid version of GoogleColab is geographically restricted, some researchers may be prevented from using RootPainter to its full potential through this platform. However, this study has demonstrated that meaningful results can be produced efficiently with the free version of GoogleColab, and while requiring more expertise, RootPainter always has the option to be run locally.

Finally, excluding the optional post-processing stage in this study would have reduced the total ‘active user time’ by 6.2 h each for Models 1 and 2, increasing the efficiency of RootPainter to 6 and 25 times faster than manual annotation for these models, respectively (table 2).

### 4.5. Improving the accuracy of RootPainter

Using smaller images may increase segmentation accuracy due to mitigation of class balance issues; large background-to-foreground ratios are known challenges for convolutional neural network model training [98]. This may be reflected in the smaller standard deviation for the average area error of Mini (0.45 ± 0.86 cm^2^) as predicted by Model 2 within smaller images, than for Magnus (−0.06 ± 2.87 cm^2^) as predicted by Model 1 . Training strategies may, therefore, require adaptation for datasets with significant class imbalances to ensure adequate model performance is achieved [44,94].

The post-processing (i.e. exclusion of obvious segmentation anomalies) completed in this study aimed to improve the accuracy of results from Models 1 and 2. Of the 452 images used in manual validation, five segmentations (including ‘16 April 2019 22.09’; electronic supplementary material, figure S7) had been removed during post-processing of Model 1. This caused no improvement in the precision, recall, dice score and accuracy of Model 1, to two decimal places. Therefore, the post-processing stage may not be necessary in future studies.

Ultimately, the accuracy of a RootPainter model depends on the quality of user corrective annotations, and whether the training images are sufficiently representative of the subject of interest. This study did not investigate the extent to which model characteristics were dependent on the annotator used. As corrective annotations are user-dependent, they may have a large influence on final model performance. Therefore, a clear definition of stopping criteria is important to increase the reproducibility of results. Generally, increases in model accuracy are expected as the amount of labelled data is increased [99], meaning that extended interaction/annotation in RootPainter will typically lead to better model performance. As manual annotations (including corrective annotations) incur some error due to ambiguity on the boundary of subjects and partial volume issues, diminishing returns in accuracy from continued annotation are seen towards the end of model training (figure 6). Therefore, accepting small inherent errors in segmentations is essential to maintaining efficiency in RootPainter studies.

### 4.6. Reliability of metric calculations in RootPainter

The corrective annotation metrics calculated within RootPainter during training overestimated precision, recall, dice score and accuracy by 0.02–0.04 compared with values from external manual validation for Model 1 (table 3). Accounting for this overestimation, the corrective metrics values for Models 2–4 still fall within the classification of successful models [4,9,17,35,41]. The discrepancy between calculations may result from images with regions of high uncertainty as during corrective annotation users can leave ambiguous errors as unclassified, conversely during manual annotation the user was forced to classify with certainty each pixel of an image. If this potential error is considered, using the corrective annotation metrics within RootPainter may negate the need for time-consuming manual validation in future studies. However, this decision should be left to users’ discretion, and it may be advised to complete manual validation when developing a model for a new species.

The overall reliability of corrective metrics calculations within RootPainter (table 3) allows identification of when the user can stop training and accurate model performance is achieved. This was trialled to success with Model 4, thus providing a possible mechanism to reduce subjectivity in training cessation across RootPainter users. However, RootPainter’s metric calculations can be skewed by imperfect user corrections. For example, in the early stages of Model 1 training the extensive background pixels were not fully correctively annotated, to avoid overwhelming the algorithm, resulting in incredibly high metrics at a time when segmentations are poor (figure 3). As Model 1 then improved its corrective metrics initially decreased as corrections became more thorough, before increasing again with the true accuracy of the model. This effect impacted the precision (and therefore, dice score) of the models most significantly, as extensive corrective annotation of the background dramatically increased the number of false-positive pixels detected. Metrics may also be misleading for subjects of interest more complex than lasers (electronic supplementary material, figures S5 and S6). Interestingly, the area error estimate by RootPainter continuously agrees with the visual assessments and can differentiate between good (figure 5*c*) and excellent (figure 5*b*) segmentations, suggesting their potential applicability as stopping criteria in future studies investigating species area. However, the ease of application of this stopping criteria should be considered; areas extracted from images originating from a mobile source, like ROVs, will each require independent scaling before valid assessments of model progress can be made. Therefore, it is recommended that the choice of stopping criteria should be determined by the nature of the dataset and that when complex subjects of interest are targeted a combined qualitative and quantitative stopping criteria approach is used.

### 4.7. RootPainter applications

Machine learning tools for image analysis have the potential to rapidly increase our understanding of marine species and their functions within ecosystems. In this study, RootPainter has demonstrated an aptitude for identifying and predicting the surface area of *M. lingua*, both in underwater observatory images and ROV video frames. Due to the high ratio of background-to-foreground pixels in images used, Models 1–3 slightly overestimated sponge area (figure 4; table 4). This error is very small and insignificant to the intended purposes of Models 1 and 2, investigating relative changes in the predicted sponge area for Magnus and Mini over time. Conversely, to estimate sponge cover or biomass, as is the purpose of Model 3, a small and consistent error in predicted area is important. While this is difficult to achieve with mobile ROV video frames, the average area error of 7.09 ± 52.97 cm^2^ for Model 3 is sufficiently small that ecological conclusions can be drawn if it is taken into consideration. The large s.d. of this area error does not represent model bias disproportionately affecting larger or smaller sponges as there is a strong correlation between the user corrected and RootPainter predicted areas for Model 3 (figure 6*d*). Finally, the ability of Model 3 to distinguish between the sponges *M. lingua* and *Geodiia* spp. (figure 5*c*), that can appear similar depending on observation conditions, confirms that the model is reliable for the investigation of ecological questions pertaining to a given species.

Results from Models 1 and 2 demonstrated the use of RootPainter to investigate temporal changes in species behaviour. Sponge contractions have previously been studied in both shallow and deep-water using manual and bespoke machine learning methods [2,33,40,41]. ‘Intrinsic’ contractions observed in shallow-water sponges probably serve to clear the aquiferous system where blocked canals may disrupt filter-feeding [100,101]. In abyssal sponges, the contracted state can be maintained for up to weeks at a time. These prolonged contractions are believed to reduce sponge filter-feeding, conserving energy as a result [2]. In this study, *M. lingua* exhibited short and frequent contractions (figure 7) consistently throughout the seasons, suggesting an alternate purpose for some sponge contractions to energy conservation and aquiferous system clearing is likely. Contractions were consistently ‘larger’ for Mini than for Magnus relative to their overall size, but the contraction rate is similar between the sponges. In April 2019, prolonged sea-star residency on Magnus coincides with an approximately 50% reduction in sponge size and a significant reduction in sponge contractions (figure 7). This energy conservation may represent a viable survival strategy for sponges during predation; contractions in Mini were unaffected during this time. Previous investigation of *M. lingua* contractions found them to be rare and asynchronous at 30 m depth [50], but frequent and correlated with salinity in one individual at 260 m depth [41]. The possibility that environmental drivers are contributing to the observed behaviour of Magnus and Mini at the LoVe Observatory requires further study. This may elucidate the purpose of the non-energy conservation contractions seen through the identification of any environmental stimuli. Variation in contractions with abiotic factors will have implications for the ecosystem services provided by deep-sea sponges, especially if frequent contractions are concluded to affect filtration capacity.

The suitability of RootPainter to spatial analyses, such as investigations into species distributions, has been shown through the successful development of odels 3 and 4. Quantifying deep-sea sponge presence and surface area allows estimation of their percentage cover and/or biomass, and therefore contribution to carbon-cycling in benthic environments [53,102]. The distribution of *M. lingua* has previously been investigated at the Tisler reef through small datasets [7,10], but the determination of its variation in time and space across the reef has been prohibited by methodological limitations. Requiring just two working days, RootPainter produced results for the distribution, abundance, and size of *M. lingua* across the east of the Tisler reef. Thus, the use of machine learning tools, such as RootPainter, will be essential in the future study of spatiotemporal patterns within large image datasets.

## 5. Conclusion

RootPainter provides a viable solution to the increasing data processing needs of marine ecologists, both on time-lapse data from static underwater observatories and frames from ROV/AUV video data. Through proper training, the algorithm can efficiently produce highly accurate models, and its built-in methods to assess stopping criteria and model success reduce the need for manual validation. Additionally, regular improvements to the software continually enhance its suitability for marine image analysis; completion of the multi-annotation capabilities of RootPainter currently under development would increase the range of ecological questions that can be tackled using RootPainter. Resource limitation is not prohibitive to accessing this user-friendly software and the adaptability of models has the capability to productively link marine image analysis researchers. Moving forward, the creation of a RootPainter repository to facilitate model sharing between users has the potential to exponentially increase the rate of information extraction from marine images, and therefore, our understanding of marine organisms.

## Data Availability

The data used and models produced in this work are accessible through Pangaea [60]. Electronic supplementary material is available online [103].
